# Impaired Bone Architecture in Peripubertal Children With HIV, Despite Treatment With Antiretroviral Therapy: A Cross‐Sectional Study From Zimbabwe

**DOI:** 10.1002/jbmr.4752

**Published:** 2022-12-10

**Authors:** Cynthia Mukwasi‐Kahari, Andrea M Rehman, Mícheál Ó Breasail, Ruramayi Rukuni, Tafadzwa Madanhire, Joseph Chipanga, Lynda Stranix‐Chibanda, Lisa K Micklesfield, Rashida A Ferrand, Kate A Ward, Celia L Gregson

**Affiliations:** ^1^ Department of Infectious Diseases Epidemiology, Faculty of Epidemiology and Population Health London School of Hygiene and Tropical Medicine London UK; ^2^ The Health Research Unit Zimbabwe (THRU‐Zim) Biomedical Research and Training Institute Harare Zimbabwe; ^3^ MRC Nutrition and Bone Health Research Group University of Cambridge Cambridge UK; ^4^ Population Health Sciences Bristol Medical School Bristol UK; ^5^ Department of Clinical Research, Faculty of Infectious and Tropical Diseases London School of Hygiene and Tropical Medicine London UK; ^6^ Child and Adolescent Health Unit, Faculty of Medicine and Health Sciences University of Zimbabwe Harare Zimbabwe; ^7^ South African Medical Research Council/Wits Developmental Pathways for Health Research Unit (DPHRU), Faculty of Health Sciences University of the Witwatersrand Johannesburg South Africa; ^8^ MRC Lifecourse Epidemiology Centre University of Southampton Southampton UK; ^9^ MRC Unit, The Gambia at LSHTM Banjul Gambia; ^10^ Musculoskeletal Research Unit, Bristol Medical School University of Bristol Bristol UK

**Keywords:** ANALYSIS/QUANTIFICATION OF BONE, DISEASES AND DISORDERS OF/RELATED TO BONE, EPIDEMIOLOGY

## Abstract

HIV infection has multi‐system adverse effects in children, including on the growing skeleton. We aimed to determine the association between chronic HIV infection and bone architecture (density, size, strength) in peripubertal children. We conducted a cross‐sectional study of children aged 8 to 16 years with HIV (CWH) on antiretroviral therapy (ART) and children without HIV (CWOH) recruited from schools and frequency‐matched for age strata and sex. Outcomes, measured by tibial peripheral quantitative computed tomography (pQCT), included 4% trabecular and 38% cortical volumetric bone mineral density (vBMD), 4% and 38% cross‐sectional area (CSA), and 38% stress–strain index (SSI). Multivariable linear regression tested associations between HIV status and outcomes, stratified by sex and puberty (Tanner 1–2 versus 3–5), adjusting for age, height, fat mass, physical activity, and socioeconomic and orphanhood statuses. We recruited 303 CWH and 306 CWOH; 50% were female. Although CWH were similar in age to CWOH (overall mean ± SD 12.4 ± 2.5 years), more were prepubertal (ie, Tanner 1; 41% versus 23%). Median age at ART initiation was 4 (IQR 2–7) years, whereas median ART duration was 8 (IQR 6–10) years. CWH were more often stunted (height‐for‐age *Z*‐score <−2) than those without HIV (33% versus 7%). Both male and female CWH in later puberty had lower trabecular vBMD, CSA (4% and 38%), and SSI than those without HIV, whereas cortical density was similar. Adjustment explained some of these differences; however, deficits in bone size persisted in CWH in later puberty (HIV*puberty interaction *p* = 0.035 [males; 4% CSA] and *p* = 0.029 [females; 38% CSA]). Similarly, puberty further worsened the inverse association between HIV and bone strength (SSI) in both males (interaction *p* = 0.008) and females (interaction *p* = 0.004). Despite long‐term ART, we identified deficits in predicted bone strength in those living with HIV, which were more overt in the later stages of puberty. This is concerning, as this may translate to higher fracture risk later in life. © 2022 The Authors. *Journal of Bone and Mineral Research* published by Wiley Periodicals LLC on behalf of American Society for Bone and Mineral Research (ASBMR).

## Introduction

Improved access and earlier antiretroviral therapy (ART) initiation in children with HIV (CWH) has markedly increased survival, enabling many children to now reach puberty and adulthood.^(^ ^1^ ^)^ The global decline in HIV‐associated child and adult deaths has largely been achieved in Eastern and Southern Africa, home to 89.2% (2.5 million of 2.8 million) of the world's CWH.^(^ ^2^ ^)^ There is increasing recognition that, despite ART, HIV has adverse effects on multiple organ systems in children resulting in long‐term multisystem comorbidities. These are of growing concern as the improved survival attributable to ART means that increasing numbers of CWH are now entering adolescence and adulthood.^(^ ^3^ ^)^ In sub‐Saharan Africa (SSA), CWH are commonly underweight (weight‐for‐age *Z*‐score <−2) and/or stunted (height‐for‐age *Z*‐score <−2), with the prevalence of stunting varying from 23% to 73%.^(^ ^3^, ^4^, ^5^ ^)^ Linear growth continues through adolescence as bone accrues to achieve peak bone mass (PBM).^(^ ^6^ ^)^ PBM is a critical determinant of adult osteoporotic fracture risk;^(^ ^7^ ^)^ a 10% reduction can double fracture risk in adulthood (although this has not yet been studied in African populations).^(^ ^8^, ^9^ ^)^ The long‐term impact of exposure to both HIV infection and ART in perinatally infected children is of concern because impaired linear growth may be associated with suboptimal PBM accrual, with implications for adult fracture risk.^(^ ^10^ ^)^

We recently reported lower dual‐energy X‐ray absorptiometry (DXA)‐measured bone outcomes of total‐body less‐head (TBLH) bone mineral content (BMC) for lean mass adjusted for height (TBLH‐BMC^LBM^) and lumbar spine bone mineral apparent density (LS‐BMAD) in CWH than in children without HIV (CWOH), which was more overt in later adolescence.^(^ ^11^ ^)^ Tenofovir disproxil fumarate (TDF) exposure has been associated with low BMD in adults living with HIV.^(^ ^12^, ^13^ ^)^ Recently, among CWH, both TDF exposure and orphanhood were associated with lower TBLH‐BMC^LBM^ *Z*‐score.^(^ ^11^ ^)^

In a smaller cross‐sectional study in Zimbabwe, age at ART initiation was strongly negatively correlated with both LS‐BMAD and TBLH‐BMC^LBM^ *Z*‐scores, with a 0.13 SD reduction in LS‐BMAD found for each year that ART initiation was delayed.^(^ ^14^ ^)^ However, both these studies used DXA, which cannot differentiate trabecular from cortical bone.^(^ ^15^, ^16^ ^)^ Peripheral quantitative computed tomography (pQCT) offers an opportunity to study bone architecture, quantifying volumetric bone mineral density (vBMD) and bone size, which enables prediction of bone strength.^(^ ^17^, ^18^ ^)^ A recent South African study compared pQCT‐measured bone architecture among 172 CWH and 98 CWOH aged 7 to 14 years, reporting lower trabecular vBMD in male CWH and generally lower bone strength in CWH than in CWOH.^(^ ^19^ ^)^

We hypothesized that HIV infection would be associated with adverse effects on bone architecture, leading to compromised pQCT bone outcomes, ie, vBMD, bone size, and predicted strength (Supplemental Fig. S1). We therefore sought to compare the bone density, bone size, and predicted bone strength parameters of trabecular and cortical bone in peripubertal males and females living with and without HIV in Harare, Zimbabwe. We investigated whether any association of HIV with pQCT‐measured bone outcomes differed by pubertal stage by testing for interaction. Furthermore, among CWH, we determined the association between TDF exposure and bone outcomes.

## Materials and Methods

### Study setting

A cross‐sectional study was conducted using baseline pQCT measurements from the IMpact of Vertical HIV infection on child and Adolescent Skeletal development (IMVASK) study, as per published protocol (ISRCTN12266984).^(^ ^11^, ^20^ ^)^ CWH, established on ART for at least 2 years, were quota sampled stratified by sex and age strata (8–10, 11–13, and 14–16 years) from HIV clinics at Parirenyatwa and/or Sally Mugabe Hospitals in Harare, Zimbabwe. These are the two large public hospitals in Harare, providing HIV care services to more than 2000 children. CWOH were randomly recruited, using school registers, from three primary and three secondary schools within the same community suburbs served by the hospitals providing HIV care, and were frequency‐matched by sex and age strata. Inclusion criteria were age 8 to 16 years, living in Harare. CWH were included if they were aware of their HIV status and had been taking ART for at least 2 years. At the time of enrollment into the study, CWOH underwent HIV testing to confirm their status. Children with acute illness requiring hospitalization, those lacking guardian consent, and those who were recently diagnosed with HIV were excluded from this study.

### Study procedures

Data were collected between May 2018 and January 2020. Demographic and clinical data were collected using an interviewer‐administered questionnaire together with handheld medical records. Demographic and clinical data collected included age, sex, socioeconomic status (SES), guardianship, orphanhood, age at HIV diagnosis, age at ART initiation, ART regimen, diet, and physical activity. SES was derived using the first component from a principal component analysis^(^ ^21^ ^)^ combining details including number in household, head of household age, highest maternal and paternal education levels, household ownership, monthly household income, access to electricity, water, a flush toilet and/or pit latrine, and ownership of a fridge, bicycle, car, television, and/or radio and was split into tertiles for analysis. The International Physical Activity Questionnaire (IPAQ) short version^(^ ^22^ ^)^ classified intensity of physical activity as low (metabolic rate [MET] minutes <600 per week), moderate (MET minutes = 600 to 3000 per week), and vigorous (MET minutes >3000 per week). Diet was assessed using a tool developed for the Zimbabwean context based on a validated dietary diversity and food‐frequency tool from India and Malawi and international guidelines applicable to SSA.^(^ ^23^, ^24^ ^)^ Daily dietary calcium (Ca) intake was classified as very low (<150 mg/d), low (150–299 mg/d), and moderate (300–450 mg/d). Daily dietary vitamin D intake was classified as very low (<4.0 μg/d), low (4.0–5.9 μg/d), and moderate (6.0–8.0 μg/d).

Puberty was Tanner staged by a trained study nurse and/or doctor. For males, testicular volume, penile size (length and circumference), and pubic hair growth (quality, distribution, and length) were assessed. For females, breast growth (size and contour) as well as pubic hair growth and age of menarche were assessed. Testicular, breast, and penile growth were graded from I to V based on Tanner descriptions.^(^ ^25^, ^26^, ^27^ ^)^ Where there was a discordance between the stages for males and females, testicular and breast development stage, respectively, were used to assign Tanner stage. Participants were grouped into Tanner stages 1 and 2 (pre‐ to early puberty) and Tanner stages 3 to 5 (mid/late puberty). Three standing height measurements were obtained to the nearest 0.1 cm using a Seca 213 stadiometer (Hamburg, Germany), and three weight measurements were obtained to the nearest 0.1 kg with a Seca 875 weight scale, with means calculated. Equipment was calibrated annually. Using British 1990 growth references, height‐for‐age *Z*‐score <−2, weight‐for‐age *Z*‐score <−2, and low weight‐for‐height body mass index (BMI) *Z*‐score <−2 were used to define stunting, underweight, and wasting, respectively.^(^ ^28^, ^29^ ^)^ Fat mass and fat‐free soft tissue (lean) mass were measured by whole‐body DXA scan, using a Hologic QDR Wi machine with Apex software version 4.5 (Hologic Inc., Bedford, MA, USA). CD4 cell count was measured using a PIMA CD4 machine (Waltham, MA, USA). HIV viral load was measured using the GeneXpert HIV‐1 viral load platform (Cepheid, Sunnyvale, CA, USA).

### pQCT scan acquisition

Non‐dominant tibial pQCT scans were performed using an XCT 2000 (Stratec Medizintecknik, Pforzheim, Germany), with voxel size 0.5 × 0.5 mm and slice thickness 2 mm (CT scan speed 30 mm/s; scout view scan speed 40 mm/s). Tibial length was measured from the distal medial malleolus to the tibial plateau with a metal ruler. Scan sites were determined as a percentage of tibia length, with the exact position determined by scout view placement of a reference line on the growth plate or on the end plate for those with fused growth plates.^(^ ^30^ ^)^ As long bones grow in length, the growth plate moves upward and the wider metaphysis is reshaped into a diaphysis by continuous resorption by osteoclasts beneath the periosteum.^(^ ^31^ ^)^ To allow for consistency of a scan site, the reference line was placed at the growth plate in those children whose end plate and growth plate were not yet fused. vBMD was measured in mg/cm^3^ at the 4% (metaphyseal) site for trabecular bone and the 38% (diaphyseal) site for cortical bone. Bone size measures included 4% and 38% total cross‐sectional area (CSA, mm^2^) and 38% cortical thickness (mm). Predicted bone strength was measured as 38% stress–strain index (SSI, mm^3^), indicating the bending and torsional strength of bone.^(^ ^32^ ^)^ The manufacturer's software (version 6.20) was used for image processing and analysis. At the 4% tibia, CALCBD was used to calculate total CSA and trabecular vBMD. CALCBD contour mode 1 (ie, threshold algorithm) was used to exclude pixels in defined regions of interest (ROI) that fell below a threshold of 180 mg/cm^3^, peel mode 1 (ie, concentric peel) peeled away the outer 55% of the total bone CSA, leaving an inner 45% CSA considered as the trabecular region of interest. At the 38% tibial site, CORTBD was used to define cortical vBMD and area; this algorithm removes all voxels within ROIs with an attenuation coefficient below a 710 mg/cm^3^ threshold (with separation mode 1). Total CSA was defined at the 38% tibia using a 180 mg/cm^3^ threshold. Cortical thickness was calculated using a circular ring model. A phantom was scanned daily for quality assurance. Thirty participants were scanned twice, with repositioning, to assess reproducibility. Coefficients of variation were calculated from the results of the 30 rescanned participants. The short‐term precision (root mean square % CV) was 1.26% for trabecular vBMD, 0.37% for cortical vBMD, 2.08% for 4% total CSA, and 0.93% for 38% CSA. All pQCT scan slices and scout views were qualitatively graded by a single radiographer. Movement artifacts were graded 0 to 3: 0 = none; 1 = slight; 2 = medium streaking; and 3 = scan unusable. Grade 3 images were excluded from analysis.

### Ethical considerations

Trained research assistants and/or a study nurse explained the study information to participants and guardians. Guardians gave written informed consent and participants gave age‐appropriate written assent. This study was approved by the Parirenyatwa Hospital and College of Health Sciences joint research ethics committee (JREC/123/19), the Biomedical Research and Training Institute Institutional Review Board (AP150/2019), the London School of Hygiene and Tropical Medicine (17154) Ethics Committee, and the Medical Research Council of Zimbabwe (MRCZ/A2494).

### Statistical analysis

Statistical analyses were performed using Stata version 16.0 (StataCorp, College Station, TX, USA). Data were cleaned and checked for consistency and outliers. Outcomes included 4% trabecular and 38% cortical vBMD (mg/cm^3^), 4% and 38% CSA (mm^2^), 38% cortical thickness (mm), and 38% SSI (mm^4^). We used independent sample *t* tests to compare continuous data between groups and percentages and chi‐square tests for categorical data. We determined differences between CWH and CWOH, stratified by sex (given different bone accrual rates) in separate regression models, by comparing means using linear regression and generating mean differences and 95% confidence intervals. We compared an unadjusted model with a model adjusting for a priori confounders; age (years), height (cm), binary pubertal status (pre/early Tanner 1–2 versus mid/late Tanner 3–5, to maximize statistical power),^(^ ^33^ ^)^ fat mass,^(^ ^34^ ^)^ physical activity,^(^ ^35^ ^)^ SES,^(^ ^36^ ^)^ and orphanhood^(^ ^11^ ^)^ (Supplemental Fig. S1). As lean mass was co‐linear with height (correlation coefficient = 0.90, *p* < 0.001), lean mass adjustment was not made to avoid collinearity. If lean mass was included in linear regression, the variance inflation factors exceeded a value of 8. We assessed modification of the association of HIV with bone outcomes by pubertal stage by incorporating a binary interaction term for puberty (pre/early versus mid/late). In further analyses, restricted to CWH, we compared those who were exposed to TDF with those who were not. To account for missing data assumed missing at random, multiple imputation by chained equations (with 7 imputed data sets), allowing for imputation of categorical and continuous data simultaneously was performed.^(^ ^37^ ^)^ Imputation models included all pQCT bone outcomes, variables associated with missingness (Tanner stage only), and factors determined in complete case analysis to be associated with HIV (fat mass, SES, orphanhood, physical activity, and sex). Imputation models were run on males and females combined, with regression analysis models using the imputed data run stratified by sex.

## Results

### Characteristics of the study population

**Fig. 1:**
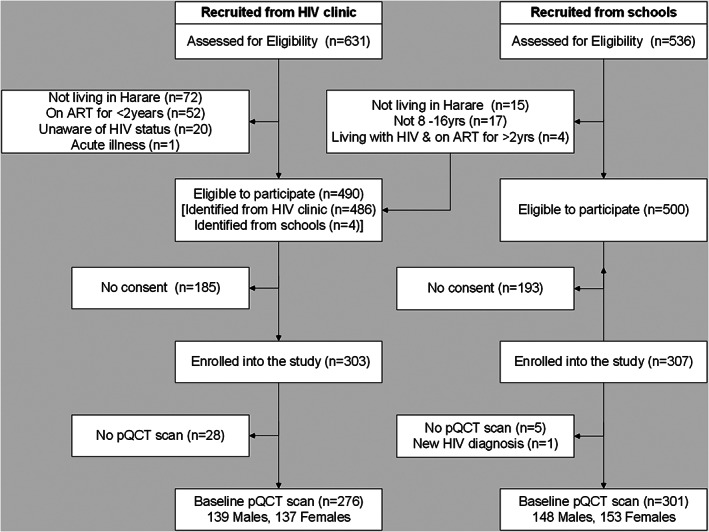
Flow diagram to show participants included in the peripheral quantitative computed tomography (pQCT) study. Figure shows participants enrolled and included in the study data analysis. All enrolled children were included in the final analysis, unless withdrawn from the study. Missing data were estimated by multiple imputation. ART = antiretroviral therapy.

The study recruited 610 participants, of whom 578 (94.8%) (276 with and 302 without HIV) had a pQCT scan (Fig. 1). One school participant was excluded because they were newly diagnosed with HIV. Participants who did not have a pQCT scan were similar to those with a pQCT scan in terms of sex, pubertal status, SES, and calcium and vitamin D intake, but children without a pQCT scan were more likely to be living with HIV and to report lower levels of physical activity compared with those who had a scan (Supplemental Table S1).

Of the participants with a pQCT scan, CWH were similar in age to those without HIV. However, CWH were more likely to be in Tanner stages 1 and 2 compared with their uninfected counterparts; this was the case for both males (67.6% versus 52.3%; *p* = 0.009) and females (55.2% versus 39.0%; *p* = 0.005) (Table 1). Height increased with Tanner stage in both CWH and CWOH (Supplemental Table S1). Compared with children without HIV, CWH were mean 8.0 cm (males) and 6.9 cm (females) shorter, and 3.7 kg (males) and 6.9 kg (females) lighter (*p* < 0.001 for all), with corresponding lower fat and lean mass.

**Table 1:** Demographic and Anthropometric Characteristics, by HIV Status, in Male and Female Children and Adolescents

Clinical characteristics	Males (*n* = 303)				Females (*n* = 306)			
	*n*	CWH (*n* = 152)	CWOH (*n* = 151)	*p* Value*	*n*	CWH (*n* = 151)	CWOH‐(*n* = 155)	*p* Value*
Age (years)								
Mean (SD)	303	12.5 (2.5)	12.4 (2.5)	0.773	306	12.4 (2.6)	12.6 (2.5)	0.518
Age group (years)								
8–10	303	52 (34.2)	50 (33.1)	0.920	306	50 (33.1)	48 (31.0)	0.890
11–13		52 (34.2)	50 (33.1)			50 (33.1)	51 (32.9)	
14–16		48 (31.6)	51 (33.8)			51 (33.8)	56 (36.1)	
Tanner stage (%)								
Tanner 1	292	57 (40.1)	45 (30.0)	0.026	299	60 (41.4)	25 (16.2)	<0.001
Tanner 2		39 (27.5)	34 (22.7)			20 (13.8)	35 (22.7)	
Tanner 3		22 (15.5)	24 (16.0)			33 (22.8)	29 (18.8)	
Tanner 4		19 (13.4)	43 (28.7)			25 (17.2)	49 (31.8)	
Tanner 5		5 (3.5)	4 (2.7)			7 (4.8)	16 (10.4)	
Socioeconomic status (%)								
Low, Tertile 1	303	54 (35.5)	38 (25.2)	0.121	306	61 (40.4)	50 (32.3)	0.014
Middle, Tertile 2		51 (33.6)	54 (35.8)			54 (35.8)	44 (28.4)	
High, Tertile 3		47 (30.9)	59 (39.1)			36 (23.8)	61 (39.4)	
Orphan status (%)								
Not an orphan	294	84 (58.3)	139 (92.7)	<0.001	299	83 (56.8)	144 (94.1)	<0.001
One parent alive		53 (36.8)	7 (4.7)			52 (35.6)	7 (4.6)	
Orphan		7 (4.9)	4 (2.7)			11 (7.5)	2 (1.3)	
Physical activity in METS (%)								
Low, <600	303	71 (46.7)	51 (33.8)	0.048	306	77 (51.0)	63 (40.6)	0.028
Moderate, 600–3000		35 (23.0)	50 (33.1)			42 (27.8)	38 (24.5)	
High, >3000		46 (30.3)	50 (33.1)			32 (21.2)	54 (34.8)	
Calcium intake (mg) (%)								
<150 mg	303	67 (44.1)	69 (45.7)	0.848	306	68 (45.0)	67 (43.2)	0.951
150–299 mg		30 (19.7)	32 (21.2)			32 (21.2)	34 (21.9)	
300–449 mg		55 (36.2)	50 (33.1)			51 (33.8)	54 (34.8)	
Vitamin D intake, μg (%)								
<4.0 μg	303	24 (15.8)	18 (11.9)	0.535	306	16 (10.6)	19 (12.3)	0.422
4.0–5.99 μg		99 (65.1)	99 (65.6)			106 (70.2)	98 (63.2)	
6.0–7.9 μg		29 (19.1)	34 (22.5)			29 (19.2)	38 (24.5)	

Anthropometry								
Height (cm)								
Mean (SD)	301	139.7 (12.6)	147.7 (15.1)	<0.001	306	140.4 (13.1)	147.3 (11.8)	<0.001
Height‐for‐age *Z*‐score								
Mean (SD)	301	−1.8 (1.2)	−0.6 (1.0)	<0.001	306	−1.5 (1.1)	−0.5 (1.1)	<0.001
Stunting (%)								
HAZ <–2, %	301	55 (36.7)	10 (6.6)	<0.001	306	40 (26.5)	12 (7.7)	<0.001
Weight (kg)								
Mean (SD)	303	35.5 (17.5)	39.2 (14.5)	0.053	306	36.1 (13.5)	43.0 (15.8)	<0.001
Weight‐for‐age *Z*‐score (mm)								
Mean (SD)	303	−1.2 (1.2)	−0.7 (1.0)	<0.001	306	−1.3 (1.1)	−0.3 (1.1)	<0.001
Underweight (%)								
WAZ <–2, %	303	50 (32.9)	15 (10.0)	<0.001	306	30 (19.9)	8 (5.2)	<0.001
Body mass index (kg/cm^2^)								
Mean (SD)	303	16.5 (1.5)	17.2 (2.2)	0.004	306	17.4 (2.7)	18.9 (3.6)	<0.001
BMI‐for‐age *Z*‐score								
Mean (SD)	303	−0.8 (0.9)	−0.5 (1.0)	0.007	306	−1.5 (1.1)	−0.5 (1.1)	<0.001
Wasting (%)								
BAZ <–2, %	303	11 (7.3)	16 (10.7)	0.313	306	5 (3.2)	12 (8.0)	0.071
Fat mass (Kg)								
Mean (SD)	288	6.8 (1.8)	8.3 (3.1)	<0.001	287	9.5 (4.3)	13.3 (6.1)	<0.001
Lean mass (Kg)								
Mean (SD)	288	26.2 (6.7)	30.1 (9.3)	<0.001	287	26.0 (6.9)	29.2 (7.3)	<0.001

HIV characteristics								
Time since HIV diagnosis (years)								
Mean (SD)	152	8.7 (2.7)			151	8.6 (2.5)		
Age at HIV diagnosis (years)								
Mean (SD)	152	3.8 (3.1)			151	3.9 (3.3)		
Duration on ART (years)								
Mean (SD)	152	8.0 (2.7)			151	7.8 (2.5)		
Age at ART initiation (years)								
Mean (SD)	152	4.5 (3.1)			151	4.7 (3.4)		
Age at TDF initiation (years)								
Mean (SD)	152	10.1 (3.8)			151	10.6 (3.2)		
Current use of TDF								
Yes	152	28 (18.4)			151	35 (23.2)		
Ever‐used TDF								
Yes	152	50 (32.9)			151	52 (34.4)		
Years of exposure to TDF								
None	152	102 (67.1)			151	99 (65.6)		
<4		30 (19.7)				32 (21.2)		
>4		20 (13.2)				20 (13.2)		
Viral load in copies/mL (%)								
<1000	135	106 (78.5)			133	106 (79.7)		
≥1000		29 (21.5)				27 (20.35)		
CD4 count in cells/μL (%)								
<200	148	4 (2.7)			140	4 (2.9)		
200–499		30 (20.3)				20 (14.3)		
≥500		114 (77.0)				116 (82.9)		

pQCT bone outcomes								
Trabecular density (mg/cm^3^)								
Mean (SD)	287	197.5 (40.0)	210.1 (40.9)	0.009	290	202.0 (33.7)	213.8 (34.1)	0.003
Cortical density (mg/cm^3^)								
Mean (SD)	287	1068.6 (39.2)	1070.8 (34.0)	0.616	290	1099.6 (38.7)	1094.9 (46.6)	0.354
4% total cross‐sectional area (mm^2^)								
Mean (SD)	287	670.1 (186.6)	772.6 (239.2)	<0.001	290	667.3 (174.5)	721.1 (193.6)	0.014
38% total cross‐sectional area (mm^2^)								
Mean (SD)	287	317.6 (70.1)	367.6 (83.6)	<0.001	290	302.3 (59.2)	348.4 (66.1)	<0.001
Cortical thickness (mm)								
Mean (SD)	287	3.6 (0.6)	3.8 (0.6)	0.066	290	3.6 (0.6)	3.6 (0.6)	0.807
Stress–strain index (mm^3^)								
Mean (SD)	287	979.8 (316.8)	1181.5 (394.1)	<0.001	290	922.8 (275.4)	1093.4 (325.9)	<0.001

Data presented are unadjusted.

CWH = children living with HIV; CWOH = children living without HIV; BMI = body mass index; ART = antiretroviral therapy; TDF = tenofovir disoproxil fumarate.

* *p* values for categorical variables were calculated using the chi‐square test; *p* values for continuous variables were calculated using the *t* test for 2 independent samples.

Overall CWH had been diagnosed with HIV when aged 3.9 ± 3.2 (mean ± SD) years and started on ART at a median age of 3.7 (IQR 1.8–6.9) years, so that at the time of participation in this study, median ART duration was 8.1 (IQR 6.2–9.5) years. The majority (79%) had a suppressed viral load (<1000 copies/mL), and only 2.3% had a CD4 count <200 cells/mm^3^. Among CWH 21% (*n* = 63/303) were taking TDF at the time of the study. However, 33.6% (*n* = 102) reported ever using TDF as part of their ART regimen, of whom 13.2% (*n* = 40) reported taking it for more than 4 years. The average age at TDF initiation was 10 years.

CWH were more likely to have a lower SES than children without HIV. Compared with children without HIV, both male and female CWH were more likely to be orphans or to have only one surviving parent (Table 1). In both sexes, physical activity levels were lower in CWH compared with those without HIV. In both males and females, dietary calcium and vitamin D intakes were similar in those with and without HIV, with the majority having a low or very low calcium intake (Table 1).

### pQCT‐measured bone outcomes, stratified by sex

#### Bone density

In unadjusted analyses, trabecular vBMD was 12.6 mg/cm^3^ (6.2%) and 11.7 mg/cm^3^ (5.2%) lower in male and female CWH than in children without HIV, whereas no such differences were found for cortical vBMD (Table 2). However, adjustment for a priori confounders completely attenuated these vBMD differences. Each of the variables included in the models had a small incremental effect on the association between HIV and each of the pQCT bone outcomes. However, adjusting for height attenuated more of the effect of HIV on trabecular and cortical density than the other covariates.

**Table 2:** Differences in pQCT‐Measured Tibial Bone Outcomes in Children Living With and Without HIV Before and After Adjustment

Males	Unadjusted model (*n* = 303)		Adjusted model (*n* = 303)	
Bone density	MD (95% CI)	*p* Value	MD (95% CI)	*p* Value
4% trabecular density (mg/cm^3^)	−12.6 (−22.1, −3.1)	0.010	−7.5 (−18.5, 3.5)	0.179
38% cortical density (mg/cm^3^)	−2.3 (−11.2, 6.5)	0.604	−1.4 (−11.8, 9.1)	0.796

Bone size				
4% total CSA (mm^2^)	−93.4 (−144.7, −42.2)	<0.001	−22.1 (−54.8, 10.6)	0.184
38% total CSA (mm^2^)	−35.6 (−66.0, −5.3)	0.021	0.4 (−24.9, 25.7)	0.975
38% cortical thickness (mm)	−0.1 (−0.3, 0)	0.063	0 (−0.1, 0.1)	0.973

Bone strength				
38% stress–strain index (mm^3^)	−189.1 (−270.1, −108.1)	<0.001	−40.2 (−95.1, 14.6)	0.150

Females	Unadjusted model (*n* = 306)		Adjusted model (*n* = 306)	
Bone density	MD (95% CI)	*p* Value	MD (95% CI)	*p* Value
4% trabecular density (mg/cm^3^)	−11.7 (−19.9, −3.6)	0.005	−7.3 (−17.7, 3.07)	0.167
38% cortical density (mg/cm^3^)	4.1 (−6.3, 14.4)	0.441	8.8 (−0.6, 18.1)	0.068

Bone size				
4% total CSA (mm^2^)	−50.7 (−92.8, −8.6)	0.019	17.1 (−18.0, 52.2)	0.338
38% total CSA (mm^2^)	−41.1 (−55.2, −27.0)	<0.001	−6.2 (−18.7, 6.4)	0.336
38% cortical thickness (mm)	0 (−0.2, 0.1)	0.817	0.2 (0.1, 0.4)	<0.001

Bone strength				
38% stress–strain index (mm^3^)	−156.0 (−226.0, −86.0)	<0.001	16.6 (−37.4, 70.6)	0.546

MD = mean difference; CI = confidence interval. Adjusted for age (years), height (cm), pubertal status, fat mass, physical activity, socioeconomic status and orphanhood. MD (95% CI) with children without HIV as the reference group, such that negative values mean that those with HIV have lower values than those with HIV. All pQCT variables, Tanner stages, and orphanhood were estimated by multiple imputation.

#### Bone size

CWH (both males and females) had smaller metaphyseal and diaphyseal tibial bone size (CSA) than CWOH (Table 2). These size differences were largely explained by adjustment. Differences in cortical thickness were only evident between females with and without HIV after accounting for age, height, and puberty, after which females with HIV appeared to have thicker cortices than females without HIV.

#### Bone strength

In both males and females, before any adjustment, SSI was lower in CWH than those without HIV; however, this was explained by adjustment for age, height, and puberty (Table 2).

### pQCT‐measured bone outcomes, stratified by sex and puberty

We next investigated the potential interaction between HIV infection and pubertal stage (pre/early [Tanner 1–2] versus mid/late [Tanner 3–5]) on bone outcomes.

#### Bone density

**Fig. 2:**
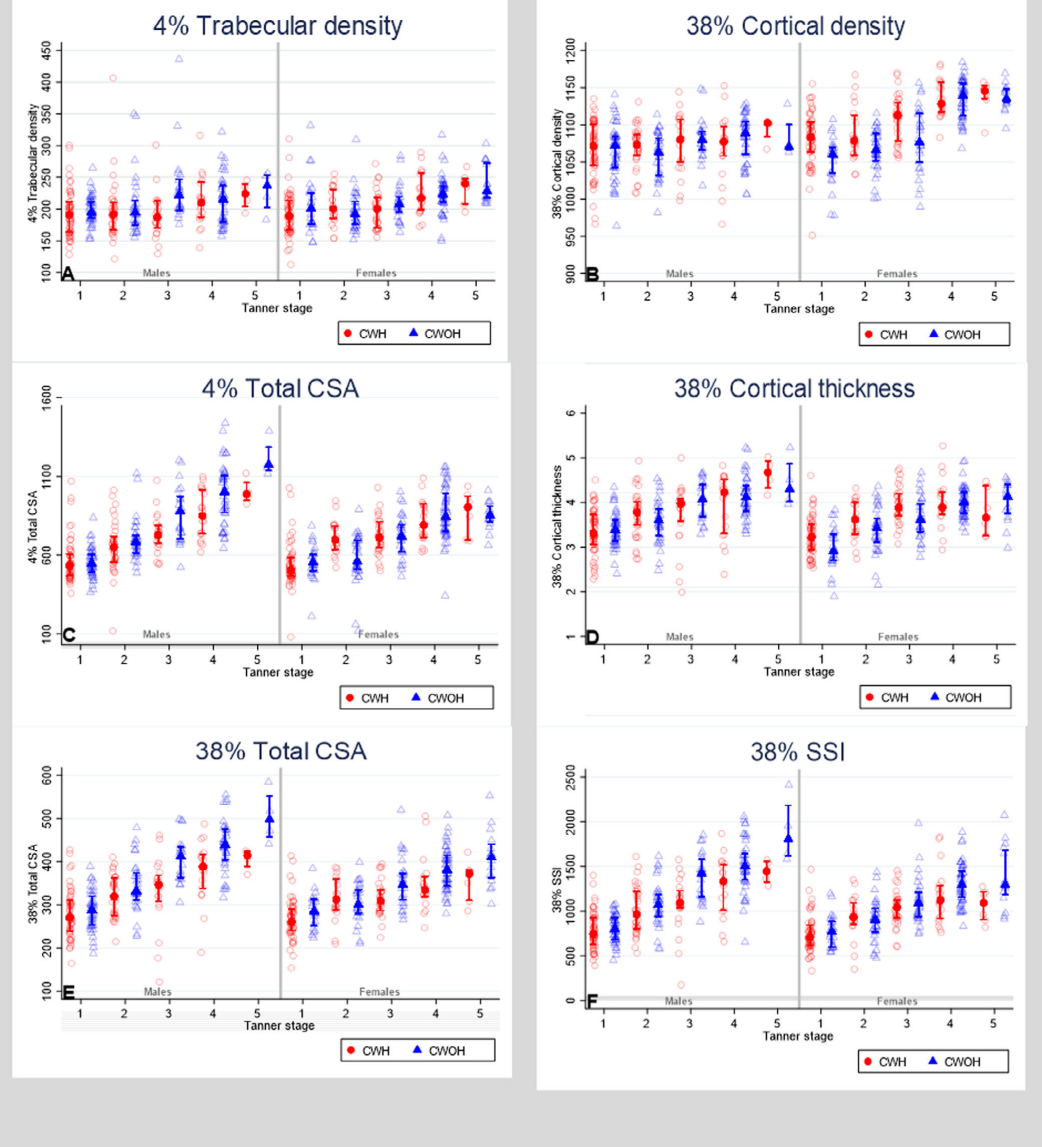
Unadjusted comparison of peripheral quantitative computed tomography (pQCT)–measured bone outcomes between children living with and without HIV infection by sex and pubertal status. The marker represents the median and the bars represent interquartile range. Data presented are unadjusted. CWH = children with HIV; CWOH = children without HIV; CSA = cross‐sectional area; SSI = stress–strain index.

Differences in trabecular vBMD between children with and without HIV were similarly small, in both pre/early and mid/later puberty, with no evidence of interaction for males or females (Fig. 2 and Table 3). However, among girls, both before and after adjustment, evidence was detected for an interaction between HIV and pubertal stage, such that in the pre/early stages of puberty females with HIV had greater cortical vBMD than females without HIV, whereas no such difference was detected in the mid/later stages of puberty (Fig. 2 and Table 3).

**Table 3:** Differences in pQCT‐Measured Bone Outcomes in Children by HIV and Pubertal Status

Males	Unadjusted model (*n* = 303)			Adjusted model (*n* = 303)		
	Tanner stages 1 and 2	Tanner stages 3, 4, and 5	*p* Value	Tanner stages 1 and 2	Tanner stages 3, 4, and 5	*p* Value
Bone density	MD (95% CI)	MD (95% CI)		MD (95% CI)	MD (95% CI)	
4% trabecular density (mg/cm^3^)	−6.1 (−17.4, 5.1)	−17.8 (−33.2, −2.4)	0.225	−3.0 (−16.7, 10.6)	−15.6 (−34.5, 3.4)	0.194
38% cortical density (mg/cm^3^)	4.4 (−6.8, 15.5)	−7.5 (−21.8, 6.7)	0.192	2.2 (−10.6, 14.9)	−7.7 (−24.5, 9.2)	0.298
Bone size						
4% total cross‐sectional area (mm^2^)	−2.2 (−46.8, 42.4)	−141.5 (−214.6, −67.4)	0.002	1.9 (−42.2, 46.0)	−65.1 (−126.4, −3.7)	0.035
38% total cross‐sectional area (mm^2^)	7.4 (−33.9, 48.7)	−74.1 (−99.3, −48.9)	0.001	19.9 (−37.0, 76.9)	−34.5 (−76.7, 7.7)	0.016
38% cortical thickness (mm)	0 (−0.1, 0.2)	−0.2 (−0.5, 0)	0.119	0.1 (−0.1, 0.2)	−0.1 (−0.3, 0.2)	0.369
Bone strength						
38% stress–strain index (mm^3^)	−23.5 (−98.9, 51.9)	−294.6 (−411.4, −177.7)	<0.001	14.3 (−50.2, 78.7)	−137.5 (−243.1, −32.1)	0.008

Females	Unadjusted model (*n* = 306)			Adjusted model (*n* = 306)		
	Tanner stages 1 and 2	Tanner stages 3, 4, and 5	*p* Value	Tanner stages 1 and 2	Tanner stages 3, 4, and 5	*p* Value
Bone density	MD (95% CI)	MD (95% CI)		MD (95% CI)	MD (95% CI)	
4% trabecular density (mg/cm^3^)	−5.8 (−18.0, 6.5)	−11.6 (−22.1, −1.1)	0.481	−8.0 (−22.5, 6.6)	−6.7 (−19.3, 5.9)	0.881
38% cortical density (mg/cm^3^)	23.4 (11.5, 35.3)	0.1 (−12.1, 12.2)	0.007	19.0 (5.1, 32.9)	−1.2 (−15.0, 12.6)	0.016
Bone size						
4% total cross‐sectional area (mm^2^)	16.2 (−34.6, 67.0)	−49.3 (−100.1, 1.6)	0.087	45.6 (−1.5, 92.7)	−10.5 (−57.4, 36.5)	0.073
38% total cross‐sectional area (mm^2^)	−17.9 (−34.8, −1.2)	−43.4 (−61.3, −25.7)	0.040	5.1 (−11.3, 21.6)	−17.1 (−33.7, −0.5)	0.029
38% cortical thickness (mm)	0.16 (0, 0.4)	0.01 (−0.2, 0.2)	0.278	0.3 (0.1, 0.5)	0.2 (0, 0.4)	0.362
Bone strength						
38% stress‐strain index (mm^3^)	−30.4 (−109.4, 48.6)	−170.6 (−253.6, −87.6)	0.016	81.2 (11.3, 150.9)	−46.1 (−119.3, 27.1)	0.004

MD = mean difference; CI = confidence interval. Adjusted for age (years), height (cm), fat mass, physical activity, socioeconomic status, and orphanhood. MD (95% CI) with children without HIV as the reference group, such that negative values mean that those with HIV have lower values than those without HIV. HIV*puberty (binary) *p* values for interaction are shown. All pQCT variables, Tanner stages, and orphanhood were estimated by multiple imputation.

#### Bone size

Before adjustment, clear evidence was detected to suggest that pubertal stage was modifying the effect of HIV on bone size, such that in both males and females, CWH in mid/late puberty had substantially smaller CSA than children without HIV, at the metaphysis and diaphysis, whereas this difference was not apparent in pre/early puberty (Fig. 2 and Table 3). Although adjustment accounted for some of these differences, evidence for interactions remained for all, other than for metaphyseal bone size in females (Table 3). No evidence was detected to suggest that puberty modifies the effect of HIV on cortical thickness.

#### Bone strength

In pre/early puberty, female CWH appeared to have higher bone strength than CWOH, whereas the reduced bone strength (SSI) found in CWH, compared with those without HIV, was most overt in mid/late puberty in both males and females (Fig. 2 and Table 3). After full adjustment, strong evidence persisted to indicate that puberty was modifying the effect of HIV on bone strength in both males (interaction *p* = 0.008) and females (interaction *p* = 0.004), such that HIV‐associated bone deficits appear worse toward the end of puberty (Table 3).

### pQCT bone outcomes in children with HIV, stratified by sex and exposure to TDF

In further analyses, restricted to CWH, those who were and were not exposed to TDF were similar in terms of sex, SES, physical activity, and dietary intake of calcium and vitamin D (Supplemental Table S1). However, those who were using TDF were older, taller, heavier, and were more likely to be in the later stages of puberty compared with CWH who were not using TDF. After adjustment, using TDF was associated with lower trabecular vBMD in males (Table 4). No other skeletal deficits were evident between TDF exposed versus non‐exposed groups once analyses were adjusted.

**Table 4:** Differences in pQCT‐Measured Bone Outcomes in Children Living With HIV by Exposure to Tenofovir

Males	Unadjusted (*n* = 151)		Adjusted (*n* = 151)	
Bone density	MD (95% CI)	*p* Value	MD (95% CI)	*p* Value
4% trabecular density (mg/cm^3^)	−12.9 (−27.7, 1.9)	0.088	−18.8 (−35.8, −1.8)	0.030
38% cortical density (mg/cm^3^)	14.5 (−0.6, 29.6)	0.060	10.1 (−8.0, 28.2)	0.269
Bone size				
4% total CSA (mm^2^)	109.6 (36.8, 182.5)	0.004	−2.4 (−70.6, 65.8)	0.945
38% total CSA (mm^2^)	85.8 (−31.6, 203.2)	0.151	64.9 (−77.4, 207.1)	0.369
38% cortical thickness (mm)	0.1 (−0.1, 0.4)	0.352	−0.14 (−0.4, 0.1)	0.323
Bone strength				
38% stress–strain index, (mm^3^)	122.3 (−10.0, 254.5)	0.070	−50.5 (−184.4, 83.4)	0.454

Females	Unadjusted (*n* = 152)		Adjusted (*n* = 152)	
Bone density	MD (95% CI)	*p* Value	MD (95% CI)	*p* Value
4% trabecular density (mg/cm^3^)	9.1 (−6.7, 24.9)	0.253	−5.4 (−23.7, 12.9)	0.557
38% cortical density (mg/cm^3^)	14.7 (−1.1, 30.4)	0.067	−11.5 (−28.3, 5.3)	0.177
Bone size				
4% total CSA (mm^2^)	175.1 (112.8, 237.4)	<0.001	45.1 (−24.9, 115.0)	0.204
38% total CSA (mm^2^)	47.2 (24.0, 70.4)	<0.001	5.6 (−20.4, 31.6)	0.668
38% cortical thickness (mm)	0.2 (0.0, 0.5)	0.049	−0.1 (−0.4, 0.2)	0.423
Bone strength				
38% stress–strain index (mm^3^)	226.6 (119.3, 333.9)	<0.001	27.1 (−86.5, 140.6)	0.635

MD = mean difference; CI = confidence interval. Adjusted for age (years), height (cm), pubertal status, fat mass, physical activity, socioeconomic status, and orphanhood. MD (95% CI) with those who are not using tenofovir disproxil fumarate (TDF) as the reference group, such that negative values mean that those using TDF have lower values than those who are not using TDF. All pQCT variables, Tanner stages, and orphanhood were estimated by multiple imputation.

## Discussion

We report results from the largest study to date to use pQCT in a sample of children and adolescents growing up with HIV infection in SSA. In Zimbabwe, we have shown that CWH have deficits in both bone size and strength compared with children who do not have HIV. These deficits included a 6% lower diaphyseal bone size in female CWH in the latter stages of puberty, even after accounting for body size and other confounders. Cortical thickness was greater in male CWH who are in the pre/early pubertal stage than in male CWOH. In females, cortical thickness was greater in CWH than in CWOH, regardless of pubertal status. Greater cortical thickness may contribute to the greater estimated bone strength reported in this study. In pre/early puberty, female CWH appear to have higher bone strength than CWOH. It is unclear whether this is attributable to the higher cortical density they showed at this pubertal stage. Despite CWH being shorter and lighter than CWOH, these results could be suggesting that CWH who are in pre/early puberty have adequate bone strength for their smaller stature. However, when they grow in height, the residual smaller bone size leads to reduced predicted bone strength so that a strength deficit begins to emerge in mid/late puberty. Although adjusted differences were less marked in females, reduced bone strength was still apparent in male CWH in the latter stages of puberty. These findings are of concern: adolescent pubertal growth is a crucial period for skeletal development; deficits in bone strength that manifest during later puberty are likely to persist into adulthood with implications for adult fracture risk.

To date, only two studies have reported using pQCT in children living with HIV.^(^ ^19^, ^38^ ^)^ Our results are consistent with findings from a smaller cross‐sectional pQCT study in South Africa of younger children (7 to 14 years), which suggested CWH have smaller bone size and lower predicted bone strength than CWOH, although this association was not adjusted for fat mass, physical activity, or any indicator of SES. Our larger study was able to both stratify by pubertal stage and adjust for a number of relevant confounders and showed how differences in size and strength become more pronounced in later puberty. A longitudinal Canadian study of 9‐ to 18‐year‐old CWH with diverse ethnic backgrounds reported no change in pQCT bone outcomes over 2 years.^(^ ^38^ ^)^ However, this study was small (*n* = 31 CWH) across sex and ethnic strata, suggesting insufficient power to detect change over time. Although notably, as found in our study, Canadian CWH in early puberty had higher cortical BMD compared with CWOH.^(^ ^38^ ^)^ An extensive literature has established a higher prevalence of stunting and underweight in CWH than in CWOH,^(^ ^4^, ^5^, ^39^, ^40^, ^41^, ^42^ ^)^ which has been confirmed by our study and our earlier studies in Zimbabwean children.^(^ ^5^, ^14^ ^)^ By adjusting for body size however, we have shown that the lower bone size in female CWH, especially in later puberty, is independent of lower height and weight.

This is the first study to use pQCT to examine the effects of TDF use on bone architecture in CWH. Our previous DXA findings in the same Zimbabwean cohort identified a strong association between TDF use and bone deficits, particularly affecting TBLH‐BMC^LBM^, such that children exposed to TDF for 4 or more years had on average a 0.5 SD lower TBLH‐BMC^LBM^ *Z*‐score compared with CWH who had not received TDF.^(^ ^11^ ^)^ This effect size is clinically important, as a 0.5 SD reduction in bone density increases by 50% both childhood and, if sustained, future adult fracture risk.^(^ ^11^ ^)^ Here we show, after accounting for multiple confounders, that TDF use is particularly associated with trabecular bone loss. Whether this translates to increased fracture risk at trabecular‐rich skeletal sites, such as the wrist and vertebra, is currently unknown.

Our reported pQCT results further extend our previous DXA findings in the same Zimbabwean cohort, where we found a higher prevalence of low TBLH‐BMC^LBM^ *Z*‐score, a cortical‐rich region of interest (10% versus 6%; *p* = 0.066) and LS‐BMAD *Z*‐score, a trabecular‐rich site (14% versus 6%; *p* = 0.0007) in CWH compared with their uninfected counterparts.^(^ ^11^ ^)^ Further earlier work in a smaller, slightly younger (6 to 16 years) Zimbabwean population identified similar prevalence of low LS‐BMAD and TBLH‐BMC^LBM^ in CWH (15% and 13%, respectively, compared with 1% and 3% in those without HIV).^(^ ^14^ ^)^ Although DXA‐measured areal BMD does not differentiate trabecular and cortical compartments, the lower LS‐BMAD previously reported indicates the possibility of a greater deficit in trabecular bone. This aligns with our unadjusted analysis that showed that trabecular vBMD and not cortical density was lower in CWH than in CWOH. However, this is inconsistent with our adjusted analysis, as we report no differences in both trabecular and cortical density in CWH and CWOH after full adjustment.

Pubertal delay is both a common consequence of HIV infection^(^ ^11^ ^)^ and a risk factor for poor bone growth.^(^ ^43^ ^)^ Hence, as expected, we saw a greater number of CWH were prepubertal than children without HIV. Pubertal delay could be the reason for compromised bone accrual in CWH. Without stratifying by pre/early versus mid/late puberty, we would have missed important interactions between HIV and pubertal stage. In our study, male CWH in mid/late puberty demonstrated the most adverse skeletal profile. The importance of stratifying by puberty was also demonstrated by a cross‐sectional study in the US of 7‐ to 24‐year‐olds, which similarly reported an HIV*puberty interaction on DXA‐measured spine and total body bone mineral content and density; the lower bone mass in participants with HIV was more pronounced with advancing puberty.^(^ ^44^ ^)^ However, in this study, the differences were more evident in males, whereas in our study, we demonstrated differences in both males and females. It is not clear to what extent the difference in the source population (eg, US versus Zimbabwe), study sampling (frequency‐matched based on Tanner stage, not age), and/or the age studied might account for these different findings.^(^ ^44^ ^)^

Our results suggesting females living with HIV particularly may be entering adulthood with bone‐size deficits is a concern. Women often experience periods of bone loss, eg, pregnancy,^(^ ^45^ ^)^ lactation,^(^ ^46^ ^)^ and menopause. Lactation is a known risk factor for bone loss in African women,^(^ ^46^, ^47^ ^)^ and 83% of Zimbabwean women continue to breastfeed beyond 1 year postpartum.^(^ ^48^ ^)^ Coupled with HIV infection and ongoing ART, the risk of adult osteoporosis is high. Recent data from rural South Africa identified a 37% prevalence of femoral neck osteoporosis in women aged ≥50 years living with HIV.^(^ ^49^ ^)^ Understanding fracture risk in African women living with HIV is an important research priority.

The major strength of this study is the novel use of pQCT in a large population of children in an understudied African population. The comparison with children without HIV and the fact that the study sample is likely representative of children living in Harare are study strengths. Our study has several limitations. The analysis is cross‐sectional; therefore, we cannot infer causality. Further, pQCT is not a technique used in clinical practice, which limits the ability to relate findings to routine clinical practice. We were unable to compare findings to normative pQCT reference data because there are no established normative pQCT data for children living in sub‐Saharan Africa. The study was not powered to stratify by pubertal stage; therefore, because males appeared to be transitioning through puberty more slowly than females, the study may not have included sufficient males at more advanced stages of puberty. Small numbers within Tanner stage categories limited our ability to test for interaction, as we needed to group participants in different pubertal stages based on numbers with each stage, rather than pubertal biology. Further follow‐up may be helpful in assessing both the male and female children further.

Our results suggest the effect of HIV on bone size and strength differs by pubertal status. We report deficits in bone size and strength associated with HIV infection, which are found more clearly toward the end of puberty in both male and female children. We further show, for the first time, an indication of attenuated trabecular bone accrual in those using TDF. We expect our findings to be broadly generalizable across populations living in southern Africa, where HIV prevalence is high. Hence, these findings have implications for fracture risk in adulthood. The study of fracture incidence, in people living with HIV in sub‐Saharan Africa, is now a research priority.

## Conflicts of Interest

The authors declare no conflicts of interest.

## Author Contributions

**Cynthia Mukwasi‐Kahari:** Conceptualization; data curation; formal analysis; investigation; methodology; project administration; writing – original draft; writing – review and editing. **Andrea M Rehman:** Conceptualization; data curation; formal analysis; methodology; supervision; writing – original draft; writing – review and editing. **Mícheál Ó Breasail:** Data curation; investigation; writing – review and editing. **Ruramayi Rukuni:** Data curation; investigation; project administration; writing – review and editing. **Tafadzwa Madanhire:** Data curation; formal analysis; writing – review and editing. **Joseph Chipanga:** Data curation; project administration; writing – review and editing. **Lynda Stranix‐Chibanda:** Supervision; writing – review and editing. **Lisa K Micklesfield:** Supervision; writing – review and editing. **Rashida A Ferrand:** Project administration; supervision; writing – review and editing. **Kate A Ward:** Conceptualization; data curation; methodology; resources; supervision; writing – original draft; writing – review and editing. **Celia L Gregson:** Conceptualization; methodology; project administration; resources; supervision; writing – original draft; writing – review and editing.

### Peer Review

The peer review history for this article is available at https://publons.com/publon/10.1002/jbmr.4752.

## Disclosure

The authors declare no conflicts of interest.

## Supporting information


**Appendix S1.** Supporting Information.

## Data Availability

Data sharing: The data that support the findings of this study are available on request from the senior authors. The data are not publicly available due to privacy/ ethical restrictions.
