# Supplementary material for: Impaired Bone Architecture in Peripubertal Children With HIV, Despite Treatment With Antiretroviral Therapy: A Cross‐Sectional Study From Zimbabwe
Source: J Bone Miner Res. 2022 Dec 10;38(2):248–60. doi: 10.1002/jbmr.4752 (PMC9996028; doi:10.1002/jbmr.4752)
Supplement: Supplementary file 1 — Appendix S1. Supporting Information. [file JBMR-38-248-s001.docx]

**Impaired bone architecture in peripubertal children with HIV despite treatment with anti-retroviral therapy: a cross-sectional study from Zimbabwe**

**Authors**

Cynthia Mukwasi-Kahari^1,2,10^, Andrea M Rehman^1^, Mícheál Ó Breasail^3,4^, Ruramayi Rukuni^2,5^, Tafadzwa Madanhire^1,2,10^, Joseph Chipanga^2^, Lynda Stranix-Chibanda^6^, Lisa K Micklesfield^7^, Rashida A Ferrand^2,5^, Kate A Ward^8,9^, Celia L Gregson^10^

**Supplementary materials**

1. Supplementary Table 1: Comparison of demographic and anthropometric characteristics in male and female children who did and did not have pQCT scans
2. Supplementary Table 2: Mean height of children with and without HIV, stratified by pubertal stage and sex
3. Supplementary Table 3: Comparison of demographic and anthropometric characteristics between children living with HIV by use of Tenofovir
4. Supplementary Figure 1: Hypothesised causal diagram showing the relationship between exposure, outcomes and the variables adjusted for in this paper

**Supplementary Table 1: Comparison of demographic and anthropometric characteristics in male and female children who did and did not have pQCT scans**

| **Characteristics** | | **No pQCT scan (n=32)** | **pQCT scan (n=577)** | **p value** |
| --- | --- | --- | --- | --- |
| HIV infected | Yes | 27 (84.4) | 276 (47.8) | <0.001 |
| Female sex | Yes | 17 (53.1) | 289 (50.2) | 0.881 |
| Age, years | Mean (SD) | 13.1 (2.9) | 12.4 (2.5) | 0.115 |
| Tanner Stage (%) | Tanner 1 | 10 (5.6) | 177 (31.7) | 0.811 |
|  | Tanner 2 | 6 (3.4) | 122 (21.8) |  |
|  | Tanner 3 | 5 (2.8) | 103 (18.4) |  |
|  | Tanner 4 | 10 (5.6) | 126 (22.5) |  |
|  | Tanner 5 | 1 (0.6) | 31 (5.5) |  |
| Socio-Economic Status (%) | Low, Tertile 1 | 10 (32.3) | 184 (33.3) | 0.122 |
|  | Middle, Tertile 2 | 15 (48.4) | 179 (32.4) |  |
|  | High, Tertile 3 | 6 (19.4) | 189 (34.2) |  |
| Orphan Status (%) | Not an orphan | 18 (58.1) | 432 (76.9) | 0.005 |
|  | One parent alive | 13 (41.9) | 106 (18.9) |  |
|  | Orphan | 0 (0) | 24 (4.3) |  |
| Physical Activity (%) | Low, <600 MET | 19 (57.6) | 243 (42.2) | 0.026 |
|  | Moderate, 600 -3000 MET | 11 (33.3) | 154 (26.7) |  |
|  | High, >3000 MET | 3 (9.1) | 179 (31.1) |  |
| Dietary calcium Intake (mg) | <150 mg | 16 (48.5) | 255 (44.3) | 0.431 |
|  | 150-299 mg | 4 (12.1) | 124 (21.5) |  |
|  | 300-449 mg | 13 (39.4) | 197 (34.2) |  |
| Dietary vitamin D Intake (mcg) | <4.0 mcg | 3 (9.1) | 74 (12.8) | 0.215 |
|  | 4.0 - 5.99 mcg | 19 (57.6) | 383 (66.5) |  |
|  | 6.0 - 7.9 mcg | 11 (33.3) | 119 (20.7) |  |
| Height, cm | Mean (SD) | 144.4 (13.8) | 143.8 (13.6) | 0.879 |
| Weight, kgs | Mean (SD) | 37.7 (11.7) | 37.1 (11.3) | 0.797 |
| Body Mass Index, kg/cm² | Mean (SD) | 18.0 (3.0) | 17.5 (2.8) | 0.369 |

*p values for categorical variables were calculated using the chi squared test, p values for continuous variables were calculated using the t test for 2 independent samples, SES = socioeconomic status. BMI; Body mass index, NB: Data presented in this table are unadjusted

**Supplementary Table 2: Mean height of children with and without HIV, stratified by pubertal stage and sex**

|  | **Height (cm)** | | | | | |
| --- | --- | --- | --- | --- | --- | --- |
|  | **CWOH (n=306)** | | | **CWH (n=303)** | | |
| **All** | **n** | **Mean** | **SD** | **n** | **Mean** | **SD** |
| Tanner 1 | 70 | 132.0 | 6.2 | 117 | 129.1 | 8.9 |
| Tanner 2 | 69 | 141.3 | 8.1 | 59 | 140.0 | 7.7 |
| Tanner 3 | 53 | 150.4 | 9.5 | 55 | 148.0 | 7.4 |
| Tanner 4 | 92 | 160.6 | 7.2 | 44 | 152.8 | 7.3 |
| Tanner 5 | 20 | 156.7 | 7.6 | 12 | 158.3 | 4.5 |
| **Males** | **n** | **Mean** | **SD** | **n** | **Mean** | **SD** |
| Tanner 1 | 45 | 131.8 | 5.8 | 57 | 129.8 | 10.1 |
| Tanner 2 | 34 | 142.8 | 8.2 | 39 | 139.0 | 6.7 |
| Tanner 3 | 24 | 153.7 | 10.7 | 22 | 147.0 | 7.6 |
| Tanner 4 | 43 | 163.5 | 7.3 | 19 | 153.8 | 8.6 |
| Tanner 5 | 4 | 167.6 | 3.8 | 5 | 160.9 | 3.7 |
| **Females** | **n** | **Mean** | **SD** | **n** | **Mean** | **SD** |
| Tanner 1 | 25 | 132.4 | 6.8 | 60 | 128.3 | 7.7 |
| Tanner 2 | 35 | 139.9 | 7.8 | 20 | 141.9 | 9.2 |
| Tanner 3 | 29 | 147.7 | 7.4 | 33 | 148.7 | 7.3 |
| Tanner 4 | 49 | 158.1 | 6.1 | 25 | 152.0 | 6.3 |
| Tanner 5 | 16 | 154.0 | 5.6 | 7 | 156.4 | 4.2 |

*Table to show mean height of children with and without HIV, stratified by Tanner stage and sex. CWH; children living with HIV. CWOH; children living without HIV.*

**Supplementary Table 3:** **Comparison of demographic and anthropometric characteristics between children with HIV by use of Tenofovir**

| **Characteristic** |  | **No TDF use(n=240)** | **TDF use(n=63)** | **p-value** |
| --- | --- | --- | --- | --- |
| Sex (%) | Female | 116 (48%) | 35 (56%) | 0.308 |
| Age, years | Mean (SD) | 11.9 (2.4) | 14.5 (1.7) | <0.001 |
| Tanner stage | Tanner 1 | 106 (47%) | 11 (18%) | <0.001 |
|  | Tanner 2 | 49 (22%) | 10 (16%) |  |
|  | Tanner 3 | 40 (18%) | 15 (25%) |  |
|  | Tanner 4 | 26 (12%) | 18 (30%) |  |
|  | Tanner 5 | 5 (2%) | 7 (11%) |  |
| Socio-Economic Status (%) | Low, Tertile 1 | 89 (37%) | 26 (41%) | 0.581 |
|  | Middle, Tertile 2 | 82 (34%) | 23 (37%) |  |
|  | High, Tertile 3 | 69 (29%) | 14 (22%) |  |
| Orphan Status (%) | Not an orphan | 141 (62%) | 26 (42%) | 0.003 |
|  | One parent alive | 71 (31%) | 34 (55%) |  |
|  | Orphan | 16 (7%) | 2 (3%) |  |
| Physical Activity in METS, (%) | Low, <600 | 121 (50%) | 27 (43%) | 0.264 |
|  | Moderate, 600 -3000 | 56 (23%) | 21 (33%) |  |
|  | High, >3000 | 63 (26%) | 15 (24%) |  |
| Calcium Intake in mg, (%) | <150 mg | 106 (44%) | 29 (46%) | 0.943 |
|  | 150-299 mg | 50 (21%) | 12 (19%) |  |
|  | 300-449 mg | 84 (35%) | 22 (35%) |  |
| Vitamin D Intake in mcg, (%) | <4.0 mcg | 32 (13%) | 8 (13%) | 0.731 |
|  | 4.0 - 5.99 mcg | 160 (67%) | 45 (71%) |  |
|  | 6.0 - 7.9 mcg | 48 (20%) | 10 (16%) |  |
| Height, cm | Mean (SD) | 137.6 (12.4) | 149.1 (9.0) | <0.001 |
| Weight, Kgs | Mean (SD) | 34.3 (16.7) | 41.2 (9.4) | 0.002 |
| BMI | Mean (SD) | 16.6 (2.0) | 18.4 (2.6) | <0.001 |

*p values for categorical variables were calculated using the chi squared test, p values for continuous variables were calculated using the t test for 2 independent samples, SES = socioeconomic status. BMI; Body mass index, NB: Data presented in this table are unadjusted

**Supplementary Figure 1: Hypothesised causal diagram showing the relationship between exposure, outcomes and the variables adjusted for in this paper**


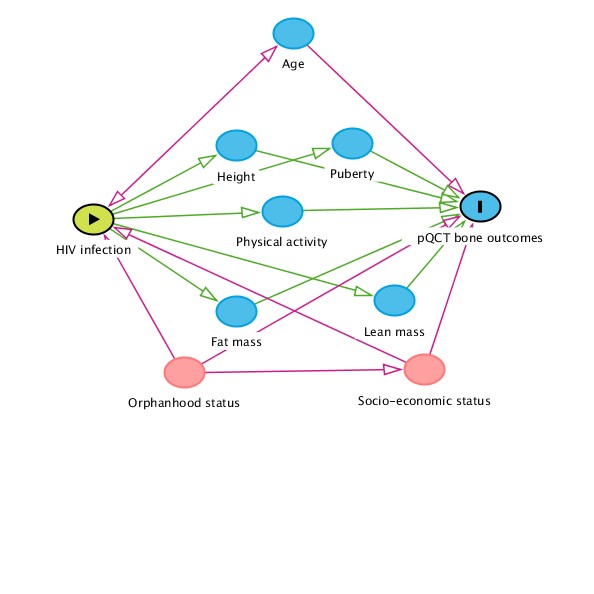


*Minimal sufficient adjustment sets for estimating the total effect of HIV infection on pQCT bone outcomes requires adjusting for age, orphanhood status and socio-economic status*
